# Inclusion of dry or wet fiber plus solubles or high protein distillers grains to replace flint corn, whole cottonseed, and soybean meal in diets for finishing Nellore bulls

**DOI:** 10.1093/tas/txaf079

**Published:** 2025-06-13

**Authors:** Silvio L Antunes, Djonatan Machado, Murilo A P Meschiatti, Isaque F Vicci, Vinícius N Gouvêa, Luis O Tedeschi, James C MacDonald, Galen E Erickson, Flávio A P Santos

**Affiliations:** Department of Animal Science, “Luiz de Queiroz” College of Agriculture, University of São Paulo, Piracicaba, SP 13418-900, Brazil; Department of Animal Science, “Luiz de Queiroz” College of Agriculture, University of São Paulo, Piracicaba, SP 13418-900, Brazil; Department of Animal Science, “Luiz de Queiroz” College of Agriculture, University of São Paulo, Piracicaba, SP 13418-900, Brazil; Department of Animal Science, University of Nebraska, Lincoln, NE 68583-0908, USA; Texas A&M AgriLife Research, Amarillo, TX 79106, USA; Department of Animal Science, Texas A&M University, College Station, TX 77843-2471, USA; Department of Animal Science, Texas A&M University, College Station, TX 77843-2471, USA; Department of Animal Science, University of Nebraska, Lincoln, NE 68583-0908, USA; Department of Animal Science, University of Nebraska, Lincoln, NE 68583-0908, USA; Department of Animal Science, “Luiz de Queiroz” College of Agriculture, University of São Paulo, Piracicaba, SP 13418-900, Brazil

**Keywords:** beef cattle, byproducts, corn, feedlot, protein, starch

## Abstract

The objective was to evaluate the inclusion of three corn ethanol byproducts in finishing cattle diets: high protein distillers grain (HPDG) was used as a source of protein in the diet to entirely replace whole cottonseed and soybean meal, dry fiber plus solubles (DFS) and wet fiber plus solubles (WFS) were used as energy-protein sources in the diet to replace a portion of the corn and entirely replace whole cottonseed and soybean meal from the conventional diet. Diets were formulated to be isonitrogenous with all diets containing 15% corn silage, 12% soybean hulls, 0.55 to 0.85% urea, and 1.80% mineral and vitamin supplements on dry matter (DM) basis. The treatments were (% DM): 1) control (CON): 8% whole cottonseed, 5% soybean meal, and 57.6% ground corn; 2) HPDG: 10.3% HPDG and 60.35% ground corn; 3) DFS: 30% DFS and 40.35% ground corn; 4) WFS: 30% WFS and 40.35% ground corn. Pre-planned contrasts included CON diet *vs*. HPDG diet, CON diet *vs*. DFS diet, and CON diet *vs*. WFS diet. In Exp. 1, 258 Nellore bulls were blocked by initial shrunk body weight (BW; 423 kg ± 36.6 kg), allocated into 44 pens in a randomized complete block design, and fed for 102 d. The average daily gain was greater (*P* = 0.02) for DFS than CON. Final BW (*P* *=* 0.02), hot carcass weight (HCW; *P* < 0.01), and *Longissimus* muscle area (*P* = 0.03) were greater for DFS than CON. In Exp. 2, 4 ruminally cannulated Nellore steers (initial BW = 389 ± 37 kg) were used in a Latin square design. Steers fed the WFS diet had less (*P* = 0.01) DMI than CON steers. The ether extract intake was greater (*P* = 0.02) for DFS and tended (*P* = 0.09) to be greater for WFS than CON, but HPDG did not differ from CON (*P* = 0.22). Steers fed DFS diets had 39.9% greater neutral detergent fiber intake (*P* < 0.01) than CON. Digestibility of DM was less (*P* < 0.01) for DFS than CON. Feeding HPDG and DFS decreased (*P* < 0.01) the molar proportion of rumen acetate, increased (*P* < 0.01) rumen propionate, and decreased acetate:propionate ratio (*P* < 0.01) compared to CON. Ruminal pH was not affected (*P* ≥ 0.82) by treatments, while ruminal ammonia nitrogen was greater for CON (*P* ≤ 0.01) compared with HPDG, DFS, and WFS. Feeding HPDG was a viable protein supplement alternative to sources like whole cottonseed and soybean meal in finishing diets. Feeding WFS and DFS improved cattle performance by displacing a portion of the flint corn and entirely replacing whole cottonseed and soybean meal.

## Teaser

Incorporating high-protein and high-fiber corn ethanol byproducts in finishing diets that include ground flint corn grain offers a viable alternative to traditional sources of protein and/or energy for feedlot cattle.

## INTRODUCTION

Traditional ethanol byproducts such as dried distillers grains (DDG), DDG plus solubles (DDGS), wet distillers grains (WDG), WDG plus solubles (WDGS), and modified distillers grains plus solubles (MDGS) have been extensively studied and used in commercial feedlot diets in the USA (Klopfenstein et al., 2008; Bremer et al., 2011; NASEM, 2016). According to Bremer et al. (2011), WDGS, MDGS, and DDGS fed from 10 to 40% of the diet, (dry matter [DM] basis) have 130 to 150%, 117 to 128%, and 112% the feeding value of dry rolled (DRC), high-moisture dent corn, or blends of both, respectively. However, new ethanol byproducts such as dry fiber plus solubles (DFS; also referred to as dry bran plus solubles), wet fiber plus solubles (WFS; also referred to as wet bran plus solubles), and high protein distillers grains (HPDG) have become available as the technology of the ethanol plants evolves (Garland et al., 2019a, b). Removing fiber from the corn kernel prior to fermentation in the ethanol production process increases the concentration of remaining components, particularly protein, resulting in a distillers grains co-product with a nutrient profile that differs from conventional forms (Garland et al., 2019a). The literature for these new byproducts (DFS, WFS, and HPDG) is still scarce.

Garland et al. (2019a) evaluated the substitution of a traditional blend of dent corn (50DRC:50HMC), soybean meal or soy-pass, and urea with alternative byproducts DDGS, WDGS, WFS, or HPDG, which made up 40% (DM basis) of the finishing diets. Cattle feed efficiency (gain-to-feed ratio; G:F) was 9.2% greater for HPDG and 10.9% greater for WFS diets compared with the control diet, suggesting feeding values of 123 and 127% relative to the blend of corn, soybean or soy-pass, and urea. Steers fed the new byproducts (HPDG and WFS) had similar or greater performance compared to traditional byproducts DDGS and WDGS.

Corn ethanol byproducts, including the traditional DDGS and WDGS, and the new byproducts DFS, WFS, and HPDG, have been increasing in availability in South America, especially in Brazil (Lima Jr., et al., 2019). They are partially replacing traditional ingredients such as flint corn, citrus pulp, soybean hulls, whole cottonseed, cottonseed meal, and soybean meal in feedlot diets (Pinto and Millen, 2018). According to Gouvêa et al. (2016) and Marques et al. (2016), steam flaking of yellow flint corn resulted in a greater increase in grain energy content than reported for the US yellow dent corn. Based on this, one may expect that the inclusion of ethanol byproducts such as HPDG, DFS, and WFS in finishing diets would yield greater dietary energy contents for diets containing flint instead of dent corns.

Lima Jr. et al. (2019) fed increasing levels of WFS containing 33.85% crude protein (CP) in a typical Brazilian feedlot diet (Pinto and Millen, 2018) containing ground flint corn, citrus pulp, soybean meal, and urea and reported that feeding 15 and 45% of WFS in the finishing diet increased cattle G:F by 5.2 and 10.4%, diet net energy for maintenance (NE_m_) by 3.6 to 9.84%, and net energy for gain (NE_g_) by 6.29% to 14.17%. The feeding values reported for WFS were 34.6 and 33.0% greater, respectively, when compared with the replaced blends of ground flint corn, citrus pulp, soybean meal, and urea. On the other hand, feedlot finishing diets in Midwest Brazil containing blends of ground flint corn, soybean hulls, soybean meal, and whole cottonseed are prevalent (Pinto and Millen, 2018). Despite the increased inclusion in finishing diets, no information is available about the effects of including HPDG, DFS, and WFS in these diets to partially replace flint corn or totally replace whole cottonseed and soybean meal, especially on the ruminal fermentation characteristics and total tract digestibility of the nutrients.

Based on the aforementioned, we hypothesized that: a) HPDG could replace whole cottonseed and soybean meal as protein sources in finishing diets with no adverse effects on cattle growth performance and carcass traits; b) feeding DFS at 30% of diet DM would provide the same protein value but greater energy content compared to a diet containing a blend of flint corn, whole cottonseed, and soybean meal; c) feeding WFS at 30% of diet DM would provide the same protein value but greater energy content than a blend of flint corn, whole cottonseed, and soybean meal. Thus, the objectives of this study were to evaluate growth performance, carcass characteristics, nutrient digestibility, and rumen fermentation characteristics of Nellore bulls fed diets containing different ethanol byproducts (HPDG, DFS, and WFS) as a replacement for typical feedlot diets based on whole cottonseed, soybean meal, and ground corn.

## MATERIALS AND METHODS

The studies were conducted at the experimental feedlot cattle facility of the Animal Science Department of the “Luiz de Queiroz” College of Agriculture (ESALQ), University of São Paulo (USP), in Piracicaba, State of São Paulo, Brazil. All procedures using animals followed guidelines recommended by the Animal Care and Use Committee of the ESALQ/USP, protocol # 2018-13.

### Finishing Experiment

#### Animals, housing, and feeding.

Two hundred sixty-four commercial Nellore bulls from grazing systems with unknown previous nutritional and management history were used in this experiment. Bulls were vaccinated against clostridia (2 ml s.c.; Sintoxan Polivalent T, Merial Saúde Animal Ltda, Paulínia, SP, Brazil), dewormed using Albendazole sulfoxide (1 ml/50 kg s.c.; Albendathor, Tortuga Companhia Zootécnica Agrária, São Paulo, SP, Brazil) and received a 5 ml s.c./animal injection of vitamins A, D and E complex (ADE Vallée, MSD Saúde Animal Ltda, São Paulo, Brazil) at feedlot arrival. During the adaptation period, six animals were withdrawn from the experiment because they showed low intake or illness likely associated with poor adaptation to bunk feeding; thus, 258 animals were used in the performance data analysis.

The experiment was preceded by a 16-d adaptation period when the dietary concentration of sugarcane bagasse was reduced every 4 d from 20% to 15% (Step-1), 15% to 10% (Step-2), 10% to 5% (Step-3), and from 5% to 0% (Step-4; DM basis) and replaced with ground corn accordingly (DM basis) in the basal diet containing sugarcane bagasse, corn silage, ground corn, citrus pulp, soybean meal, urea, and mineral premix. Corn silage was fixed at 15% of dietary DM during the adaptation period and in the finishing diet (Tables 1 and 2). No corn byproducts were included in the basal diet during the adaptation period. After the adaptation period, bulls were individually weighed after 16 hours of feed and water withdrawal (initial shrunk body weight (BW) = 423 ± 37 kg) and given a unique ear tag. Cattle were blocked by initial shrunk BW (d 0; 11 BW blocks) and allocated to 44 feedlot pens (11 pens/treatment), 36 partially covered feedlot pens (4 × 8 m) with concrete floor, (30 pens with six bulls per pen (5.3 m^2^/bull and 0.67 m bunk space/bull) and six pens with five bulls/pen (6.4 m^2^/bull and 0.8 m bunk space/bull), and another 8 pens (7 × 12m) soil-surfaced not covered (6 bulls/pen; 14 m^2^/bull and 1.17 m bunk space/bull). Each pen shared water tanks with every other pen, and these tanks were cleaned weekly throughout the experiment. Treatments were assigned randomly to each pen type and consisted of four treatment diets with or without corn ethanol byproducts (CON, HPDG, DFS, and WFS; Table 1). Diets were formulated to meet or exceed the energy requirements of finishing Nellore bulls for 1.5 kg average daily gain (ADG) as specified by NASEM (2016) and containing a similar concentration of crude protein (CP; Table 2 and 3): control (CON) = no corn ethanol byproduct included; HPDG = 10.3% of HPDG (DM basis) replacing whole cottonseed and soybean meal in the control diet; DFS = 30% of DFS (DM basis) partially replacing a portion of the corn and replacing whole cottonseed and soybean meal in the control diet, and WFS = 30% of WFS replacing DFS.

**Table 1.** Chemical composition (% of dry matter, [DM]) of feed ingredients [corn silage (CS), ground corn (GC), soybean hulls (SBH), soybean meal (SBM), whole cottonseed (WCS), high protein distillers grains (HPDG), dry fiber plus solubles (DFS), and wet fiber plus solubles (WFS)] used in experimental diets

Item	CS	GC	SBH	SBM	WCS	HPDG	DFS	WFS
Dry matter (%, as fed)	40.7	89.0	89.8	91.6	90.0	94.5	88.5	42.4
Crude protein, %	7.90	8.50	10.4	47.0	22.5	44.3	18.2	18.2
Neutral detergent fiber, %	47.1	12.7	78.2	16.2	42.5	33.1	48.5	50.5
Ether extract, %	3.10	4.47	1.12	2.17	17.8	14.3	10.3	10.9
Ash, %	4.00	1.8	4.11	6.65	3.80	2.28	6.28	5.63
Gross energy, Mcal/kg DM	4.40	4.51	4.17	4.35	5.46	6.12	5.34	5.40

^1^Corn ethanol byproducts (DFS, WFS, and HPDG) were provided by FS Fueling Sustainability (Lucas do Rio Verde, MT, Brazil).

**Table 2.** Ingredients and chemical composition of experimental diets (dry matter [DM] basis)

Item	Control	HPDG	DFS	WFS
Ingredient, %				
Corn silage	15.0	15.0	15.0	15.0
Soybean hulls	12.0	12.0	12.0	12.0
Ground corn^1^	57.6	60.4	40.4	40.4
Whole cottonseed	8.00	-	-	-
Soybean meal	5.00	-	-	-
HPDG^2^	-	10.3	-	-
DFS^3^	-	-	30.0	-
WFS^4^	-	-	-	30.0
Urea	0.60	0.50	0.80	0.80
Mineral and vitamin supplement^5^	1.50	1.50	1.50	1.50
Sodium chloride (salt)	0.30	0.30	0.30	0.30
Analyzed composition^6^, %				
Dry matter, % as fed	75.9	76.2	75.7	59.2
Crude protein	13.1	13.6	13.8	13.8
Neutral detergent fiber	27.9	27.4	36.0	36.6
Ether extract	4.68	4.74	5.46	5.64
NE_m_, Mcal/kg^7^	1.98	1.98	1.98	2.06
NE_g_, Mcal/kg^7^	1.33	1.33	1.34	1.40

^1^Ground corn was processed through a hammer mill (ML 100-A, Lucato, Indústria e Comércio Lucato, Limeira, SP, Brazil) to achieve a mean particle size of 1.36 mm (Gouvêa et al., 2016).

^2^HPDG = high protein distillers grains.

^3^DFS = dry fiber plus solubles.

^4^WFS = wet fiber plus solubles.

^5^Custom blend containing (DM basis) 275 g/kg Ca, 10 g/kg Mg, 56 g/kg S, 2,240 mg/kg of Mn, 3,360 mg/kg of Zn, 1,120 mg/kg of Cu, 16.46 mg/kg of Co, 56 mg/kg of I, 11.2 mg/kg of Se, and 2,000 mg/kg of monensin.

^6^Based on chemical analysis of composited samples of each ingredient collected weekly throughout the experiment.

^7^The dietary expected net energy for maintenance (NE_m_) and gain (NE_g_) were estimated according to the equations proposed by NASEM (2016; solution type = empirical level) with the addition of monensin as the feed additive and based on the sum of total digestible nutrient (TDN) tabular values from each ingredient. The NASEM (2016) tabular TDN values of DDGS and WDGS were used for DFS and WFS, respectively.

**Table 3.** Ground corn particle size distribution (% of total)^1^

Pores in the size	%
> 6.0 mm	0.00
≤ 6.0 and > 3.5 mm	0.34
≤ 3.5 and > 2.0 mm	13.54
≤ 2.0 and > 1.25 mm	44.22
≤ 1.25 mm	41.9
Mean particle size, mm^2^	1.36

^1^Flint corn grain was processed through a hammer mill (ML 100-A, Lucato, Indústria e Comércio Lucato, Limeira, SP, Brazil).

^2^Grain particle distribution was determined according to Yu et al. (1998) using sieves with 6.0, 3.5, 2.0, and 1.25 mm square pores (Produtest T Model; Telastem Peneiras para Análises Ltda, São Paulo, SP, Brazil). Particles < 1.25 mm were assumed to represent a 0.625 mm square pores sieve (Gouvêa et al., 2016).

The HPDG, DFS, and WFS products were produced by FS Fueling Sustainability (Lucas do Rio Verde, MT, Brazil; trade names FS Essencial, FS Ouro, FS umido, respectively for HPDG, DFS, WFS) and shipped to the experimental feedlot cattle facility (approximately 1,700 km) in one single load for HPDG (bagged, 45 kg/bag), three loads for DFS, and four loads for WFS. Every 3 to 4 wk, new loads of DFS and WFS were received at the research facility, stored on top of a plastic tarp, and covered with another plastic tarp, protecting the feed from having direct contact with ground and rainfall. The flint corn grain was processed through a hammer mill (ML 100-A, Lucato, Indústria e Comércio Lucato, Limeira, SP, Brazil) for a mean particle size of 1.36 mm (Table 3), assayed as described by Yu et al. (1998), using sieves with 6.0, 3.5, 2.0, and 1.25 mm square pores (Produtest T Model; Telastem Peneiras para Análises Ltda., São Paulo, SP, Brazil).

Bulls were fed once daily at 1300 h throughout the experiment and had free choice access to feed and fresh water. Corn silage, WFS, and DFS were weighed into the feed-mix wagon (Totalmix 25, Casale Equipamantos, São Carlos, Brazil). Whole cottonseed, soybean hulls, soybean meal, HPDG, urea, sodium chloride, and mineral supplements were individually weighed into nylon bags using a fixed precision scale (Weightech WT1000, Weightech Equipamentos de Pesagem, Florianópolis, SC, Brazil, readability = 50.0 g) before being emptied into the feed wagon. The total mixed ration was prepared using a feed-mix wagon (Totalmix 25, Casale Equipamantos), and mixed for 5 min. The amount of fresh feed offered to each pen was adjusted daily based on the DMI of the previous day, assuming the amount of orts should not exceed 3% of the daily intake of DM/animal, based on bunk scores at 1200 h. Orts were collected daily, weighed (Weightech WT1000), sampled, and dried in a forced-air oven at 105 °C for 12 h to quantify DMI for each pen.

#### Sample collection and analyses.

At the beginning of the experiment (d 0; after the 16-d adaptation period) and at the end of the experiment (d 102), individual shrunk BW was recorded after 16-h of feed and water withdrawal, and these data were used to calculate ADG and G:F, which was calculated by dividing pen ADG by pen DMI.

Corn silage and WFS samples were collected every 3 d and dried at 105 °C for 12 h for diet DM adjustments. Samples of corn silage, ground corn, soybean hulls, whole cottonseed, soybean meal, HPDG, DFS, and WFS were collected weekly and stored at −20 °C. At the end of the experiment, feed samples were thawed, composited by feed ingredient, dried in a forced-air oven at 55 °C for 72 h, and ground using a Wiley-type mill (MA-680 Marconi Ltda, Piracicaba, SP, Brazil) through a 1-mm screen. All samples were analyzed for DM at 105 °C (method 930.15; AOAC, 1986), ash (method 942.05; AOAC, 1986), ash-corrected neutral detergent fiber (NDF; Van Soest et al., 1991, modified for Ankom 200 fiber analyzer, Ankon Technology Corp., Macedon, NY) using sodium sulfite for all samples and heat-stable α-amylase for corn samples only, acid detergent fiber (ADF; Goering and Van Soest, 1970), ether extract (method 920.85; AOAC, 1986), total N (Leco FP-528, Leco Corp., St. Joseph, MI), and gross energy (GE) using a bomb calorimeter (Model MS 10A of Reichel & Partner GmbH, German; AOAC, 2000). Crude protein (CP) was calculated multiplying N concentration by 6.25.

The calculated feed net energy (NE) concentration based on performance data was determined using the equations proposed by Zinn and Shen (1998). Energy of gain (EG) was calculated as EG = (0.0557 × BW^0.75^) × ADG^1.097^ (NRC, 1984), where EG is daily energy deposited (Mcal/d), and BW is the mean shrunk BW for the period. The equation used to calculate maintenance energy expended (EM; Mcal/d) was EM = 0.077 × BW^0.75^ (Lofgreen and Garrett, 1968). From calculated amounts of energy required for maintenance and gain, the net energy of maintenance (NE_m_) of each diet was obtained by the quadratic equation: NE_m_$=\frac{-b\pm \sqrt{b^{2}-4ac}}{2a}$, where a = −0.41 × EM, b = 0.877 × EM + 0.41 × DMI + EG, and c = −0.877 × DMI; and net energy of gain (NE_g_) of each diet was obtained by the equation: NE_g_ = 0.877 × NE_m_—0.41 (Zinn and Shen, 1998).

The expected dietary NE_m_ and NE_g_ were calculated based on the sum of NASEM (2016) tabular energy values for each feed ingredient, with the addition of monensin (2.3% increase in diet ME content; NASEM, 2016) and the ratios for observed NE_m_ to the expected NE_m_ and observed NE_g_ to the expected NE_g_ were calculated.

At the end of the feeding experiment bulls were transported 10.3 km (approximately 20 min), using commercial beef trucks, to a commercial packing plant (Frigorífico Angelelli Ltda, Piracicaba, SP), where they were slaughtered on the following day. Hot carcass weight (HCW) was obtained at the time of slaughter following evisceration and removal of kidney, pelvic, and heart (KPH) fat; dressing percentage was calculated as the ratio of HCW to final shrunk BW. Subcutaneous fat thickness and Longissimus muscle (LM) area were measured over the 12th rib after a 24 h carcass chill at 4 °C using a digital camera attached to a 10 cm high rod attached to a 15 × 20 cm rectangular steel base. The images obtained by the digital camera were interpreted by an experienced technician using the Lince software (M&S Consultoria Agropecuaria, Pirassununga, SP, Brazil).

#### Statistical analyses.

Data were analyzed using the PROC MIXED procedure of SAS (version 9.4, SAS Inst., Inc., Cary, NC) as a randomized complete block design (11 replications/treatment). Animals were blocked by initial shrunk BW, and pens were the experimental units. The statistical model included the fixed effect of treatment and the random effect of blocks (n = 11). The Kenward-Roger approximation method was used to determine the denominator degrees of freedom for testing fixed effects. All results were reported as least-square means.

If a significant ANOVA *P-*value for the main effect of treatment was observed, three pre-planned contrasts were evaluated using the CONTRAST statement: 1 = control diet *vs*. diet containing high protein distillers grains (HPDG), 2 = control diet *vs*. diet containing dry fiber plus solubles (DFS), and 3 = control diet *vs*. diet containing wet fiber plus solubles (WFS). These contrasts were selected a priori based on the primary objective of the study, which was to evaluate the effects of individual alternative diets relative to a common control. This approach avoids the increased Type I error associated with multiple pairwise comparisons and provides a more focused assessment of treatment effects. Differences among treatments were considered different when *P* ≤ 0.05, with trends noted when 0.05 < *P* ≤ 0.10.

### Metabolism Experiment

#### Animals, housing, and feeding.

Four ruminally cannulated Nellore steers (initial BW = 390 ± 37 kg) were used in a 4 × 4 Latin square design to evaluate intake, apparent total tract digestibility, and ruminal fermentation characteristics of diets used in the finishing experiment (Tables 1, 2, and 3). Steers were housed in individual pens (4 × 8m) with a solid roof and concrete floor, and the 14-d periods consisted of 10-d for adaptation to the experimental diets followed by a 4-d collection period. During the first 10-d, diets were offered for ad libitum once a day at 0800 h. During the 4-d collection period, daily feed intake was restricted to 90% of the mean feed intake observed for each animal during the previous 10-d adaptation period to ensure that the animal ingested the amount offered.

#### Sample collection and analyses.

Fecal grab samples (approximately 50 g) were manually collected directly from the rectum of each steer twice a day during the 4-d collection period for nutrient analyses and digestibility calculations (Zinn and Barajas, 1997) in a manner that all collections represented one full day. Samples were dried in a forced-air oven at 55 °C for 72 h and ground using a Wiley-type mill (MA-680 Marconi Ltda, Piracicaba, SP, Brazil) through a 1-mm screen. Equal amounts of fecal DM were mixed to obtain one representative sample for each steer for each experimental period and analyzed for DM, CP, ether extract (EE), NDF, and GE as described for the finishing experiment. During these 4-d collection period, steers were monitored every 2 hours, and the total daily fecal production was measured by collecting the feces from the concrete floor every 2 hours (Meschiatii et al., 2019) to determine total fecal output. Feces were immediately weighed and mixed after each collection, and a sample (10% of the total amount) was dried at 105 °C for 12 h for DM analysis and total fecal production calculation (animal/day). The amount of feces collected directly from the rectum of each animal for chemical analysis was added to the total feces collected from each pen for total fecal production calculation.

During the last day of the collection period (d 14), approximately 50 mL of ruminal content was collected from 3 locations in the ventral rumen as described by Danés et al. (2013) at 0, 2, 4, 6, 8, 10, 12, 16, 20 and 24 h post-feeding. Ruminal fluid samples were squeezed through 4 layers of cheesecloth, and ruminal pH was immediately measured using a portable pH meter (Digimed Model DM22, Digicrom Analítica Ltda., São Paulo, SP, Brazil). Samples of 2 ml per collection time were placed in microcentrifuge tubes and preserved using 1 ml of 8.6 M H_2_SO_4_ solution and stored at –20 °C. At the end of the experiment, ruminal fluid samples were thawed and centrifuged at 15,000 × *g* for 30 min at 4 °C. The supernatant fluid was analyzed for volatile fatty acids (VFA) by gas-liquid chromatography (Palmquist and Conrad, 1971) and for rumen ammonia nitrogen (NH_3_–N; Chaney and Marbach, 1962). The VFA concentration was determined as described by Polizel et al. (2020). Briefly, 1.8 mL of ruminal fluid was centrifuged at 15,000 × *g* for 60 min at 4 °C. Following centrifugation, 0.8 mL of the supernatant was transferred to a chromatography vial, and 0.2 mL of a 3:1 solution of metaphosphoric acid (250 mL/L) and formic acid (980 mL/L) was added. An internal standard (0.1 mL of 100 mM 2-ethylbutyric acid) was then added to each vial. VFA concentrations were determined using an Agilent 7890A gas chromatograph equipped with a flame ionization detector and a fused-silica capillary column (25 m × 320 μm i.d.) coated with 0.20 μm cyanopropyl polysiloxane. The chromatographic program consisted of three temperature stages: an initial hold at 80 °C for 1 min, a ramp to 120 °C at 20 °C/min held for 3 min, followed by a ramp to 205 °C at 10 °C/min held for 2 min. Hydrogen was used as the carrier gas at a flow rate of 1.0 mL/min, with injector and detector temperatures set at 260 °C. The NH_3_–N was analyzed using a colorimetric method adapted for a microplate analysis, with absorbance measured at 550 nm using a microplate reader (BioRad, Hercules, CA, USA) as described by Toseti et al. (2020).

#### Statistical analysis.

Data from the metabolism study were analyzed using PROC MIXED of SAS as a 4 × 4 Latin square design. The statistical model used to analyze intake and nutrient digestibility included the fixed effect of treatment and the random effects of steers and experimental periods. Steer within period was the experimental unit. Ruminal fermentation characteristics (VFA, pH, and NH_3_-N) were analyzed as repeated measures by collection time. The statistical model included the fixed effect of treatment and the random effects of steers and experimental periods. Time was the repeated measure, and the subject was animal(treatment). Different structures of the variance-covariance matrices were tested, and the variance component matrix was chosen based on the best fit for most variables using Akaike’s information criterion. Because no treatment × time interactions were detected, time was excluded from the model and treatment means were reported to simplify data interpretation. The Kenward-Roger approximation was used to determine the denominator degrees of freedom for testing fixed effects. Similarly to Exp. 1, if a significant ANOVA *P-*value for the main effect of treatment was observed, three pre-planned contrasts of interest were tested 1 = control diet *vs*. HPDG diet, 2 = control diet *vs*. DFS diet, and 3 = control diet *vs*. WFS diet. All results were reported as LS means. Differences among treatments were considered different when *P* ≤ 0.05, with trends noted when 0.05 < *P* ≤ 0.10.

## RESULTS

### Finishing Experiment

Feeding corn ethanol byproducts HPDG, DFS, and WFS did not affect DMI compared to CON (*P* ≥ 0.16; Table 4). The ADG was 9.46% greater (*P* = 0.02) only for DFS compared to CON; no other significant differences (*P* ≥ 0.44) were observed for ADG. No treatment effect was observed for G:F (*P* = 0.30). Final BW was 2.09% greater (*P* = 0.02) and HCW was 3.43% (11.0 kg) greater (*P* < 0.01) for DFS compared to CON (Table 4). No treatment effect was observed for carcass dressing percentage (*P* = 0.44). Bulls fed with DFS had greater (*P* = 0.03) LM area compared to CON. Treatments did not affect 12th rib-fat thickness or the observed NEm and NEg calculated based on growth performance data (*P* ≥ 0.18; Table 4).

**Table 4.** Effect of different corn ethanol byproducts [high protein distillers grains (HPDG), dry fiber plus solubles (DFS), and wet fiber plus solubles (WFS)] on growth performance and carcass characteristics of finishing Nellore bulls

Item	Treatments^1^				SEM^2^	Treatment *P*-value	Pre-planned contrasts *P*-value^3^		
	Control	HPDG	DFS	WFS			1	2	3
Growth performance									
Initial body weight, kg	423	423	423	423	13.9	-	-	-	-
Final body weight, kg	574	570	586	576	13.1	0.03	0.48	0.02	0.61
Average daily gain, kg	1.48	1.45	1.62	1.53	0.058	0.03	0.58	0.02	0.44
Dry matter intake, kg/d	10.8	10.4	11.2	10.3	0.369	0.06	0.30	0.24	0.16
Feed efficiency	0.139	0.140	0.144	0.149	0.005	0.30	0.85	0.37	0.09
Carcass characteristics									
Hot carcass weight, kg	321	322	332	320	7.51	0.01	0.93	<0.01	0.78
Dressing, %	56.1	56.8	56.6	55.9	0.459	0.44	0.24	0.43	0.79
* Longissimus* muscle area, cm^2^	64.9	66.5	67.8	66.6	1.31	0.07	0.21	0.03	0.19
12^th^-rib fat, mm	3.55	4.17	3.98	4.21	0.242	0.18	0.07	0.20	0.05
Observed NE, no., Mcal/kg^4^									
Maintenance	1.89	1.91	1.93	2.01	0.066	0.27	0.73	0.53	0.07
Gain	1.25	1.27	1.28	1.35	0.058	0.27	0.73	0.56	0.07

^1^Corn ethanol byproducts were provided by FS Fueling Sustainability (Lucas do Rio Verde, MT, Brazil).

^2^SEM = standard error of the mean.

^3^Pre-planned contrasts: 1 = Control *vs*. HPDG; 2 = Control *vs*. DFS; 3 = Control *vs*. WFS.

^4^Calculated using cattle growth performance data based on the equation proposed by Zinn and Shen (1998).

### Metabolism Experiment

Steers fed WFS consumed 1.47 kg of DM/d less (*P* = 0.01) than steers fed the CON diet (Table 5). No other differences (*P* ≥ 0.20) were observed for DMI. Treatments did not affect crude protein intake (*P* ≥ 0.12). As expected, based on the chemical composition of the ingredients and diets (Tables 1 and 2), EE intake was 19.44% greater (*P* = 0.02) for steers fed DFS compared to CON, and tended (*P* = 0.09) to be 12.5% less for steers fed WFS, with no difference between HPDG and CON (*P* = 0.22; Table 5). Also, as expected, steers fed DFS had 39.9% greater NDF intake (*P* < 0.01) than CON. Digestibility of DM was less (*P* < 0.01) for DFS compared to CON (Table 5). Feeding corn ethanol byproducts HPDG, DFS, and WFS did not affect the apparent total tract digestibility of CP, EE, and NDF (*P* ≥ 0.24; Table 5).

**Table 5.** Effect of different corn ethanol byproducts [high protein distillers grains (HPDG), dry fiber plus solubles (DFS), and wet fiber plus solubles (WFS)] on intake and nutrient digestibility of finishing Nellore steers

Item	Treatments^1^				SEM^2^	Treatment *P*-value	Pre-planned contrasts *P*-value^3^		
	Control	HPDG	DFS	WFS			1	2	3
Average body weight, kg	445	439	443	438	13.8	-	-	-	-
Intake, kg/d									
Dry matter	8.07	7.54	8.39	6.60	0.410	0.02	0.20	0.49	0.01
Crude protein	1.05	1.05	1.14	0.954	0.056	0.08	0.88	0.18	0.12
Ether extract	0.360	0.332	0.430	0.315	0.021	0.01	0.22	0.02	0.09
Neutral detergent fiber	2.03	1.92	2.84	2.09	0.127	<0.01	0.41	<0.01	0.64
Total tract apparent digestibility, %									
Dry matter	75.4	76.8	68.5	73.7	2.22	0.02	0.25	<0.01	0.24
Crude protein	68.2	69.9	65.7	69.9	2.76	0.20	0.33	0.24	0.36
Ether extract	89.9	88.6	87.8	87.8	1.66	0.65	0.46	0.33	0.30
Neutral detergent fiber	43.8	42.2	45.8	45.4	6.84	0.97	0.85	0.84	0.86

^1^Corn ethanol byproducts were provided by FS Fueling Sustainability (Lucas do Rio Verde, MT, Brazil).

^2^SEM = standard error of the mean.

^3^Pre-planned contrasts: 1 = Control *vs*. HPDG; 2 = Control *vs*. DFS; 3 = Control *vs*. WFS.

Treatments did not affect total rumen VFA concentration or rumen pH (*P* ≥ 0.46; Table 6). Feeding HPDG and DFS decreased the molar proportion of rumen acetate (*P* < 0.01), increased rumen propionate (*P* < 0.01), and decreased acetate:propionate ratio (*P* < 0.01) compared with CON (Table 6). No differences were observed between WFS and CON for the molar proportion of rumen acetate, propionate, or acetate:propionate ratio (*P* ≥ 0.27). The molar proportion of rumen butyrate was greater (*P* ≤ 0.05) for steers fed byproducts HPDG, DFS, and WFS compared to CON. Feeding HPDG and DFS decreased (*P* < 0.01) molar proportion of rumen isobutyrate and isovalerate compared to CON. Valerate was greater (*P* = 0.01) for steers fed DFS and tended (*P* = 0.07) to be greater in steers fed HPDG compared with CON (Table 6). Ruminal NH_3_-N concentration was greater for CON (14.8 mg/dL; *P* ≤ 0.01) compared with HPDG (6.35 mg/dL), DFS (10.5 mg/dL), and WFS (10.1 mg/dL; Table 6).

**Table 6.** Effect of different corn ethanol byproducts [high protein distillers grains (HPDG), dry fiber plus solubles (DFS), and wet fiber plus solubles (WFS)] on ruminal fermentation characteristics of finishing Nellore steers

Item	Treatments^1^				SEM^2^	Treatment *P*-value	Pre-planned contrasts *P*-value^3^		
	Control	HPDG	DFS	WFS			1	2	3
Total volatile fatty acids (VFA), mM	83.6	95.2	94.0	83.3	9.85	0.46	0.25	0.30	0.97
VFA proportion, mol/100 mol									
Acetate	61.9	56.9	57.4	61.6	1.23	<0.01	<0.01	<0.01	0.70
Propionate	24.3	28.5	28.8	23.4	1.41	<0.01	<0.01	<0.01	0.27
Butyrate	9.44	11.2	10.4	10.7	0.448	<0.01	<0.01	0.05	0.02
Isobutyrate	0.979	0.575	0.504	0.978	0.066	<0.01	<0.01	<0.01	0.99
Valerate	1.39	1.51	1.56	1.32	0.051	<0.01	0.07	0.01	0.32
Isovalerate	2.24	1.50	1.29	2.26	0.234	<0.01	<0.01	<0.01	0.89
Acetate:propionate	2.70	2.12	2.07	2.79	0.170	<0.01	<0.01	<0.01	0.39
Ruminal pH	6.59	6.65	6.65	6.60	0.188	0.99	0.82	0.84	0.98
Ruminal NH_3_-N, mg/dL	14.8	6.35	10.5	10.1	1.49	<0.001	<0.01	0.01	<0.01

^1^Corn ethanol byproducts were provided by FS Fueling Sustainability (Lucas do Rio Verde, MT, Brazil).

^2^SEM = standard error of the mean.

^3^Pre-planed contrasts: 1 = Control *vs*. HPDG; 2 = Control *vs*. DFS; 3 = Control *vs*. WFS.

## DISCUSSION

When included in feedlot diets high in dent corn (dry rolled, high moisture, or blends of both) at 10 to 40% of DM, WDGS, MDGS, and DDGS have greater energy content than the cereal grain and improve cattle performance (Klopfenstein et al., 2008; Bremer et al., 2011; DiConstanzo et al., 2015; NASEM, 2016). Garland et al. (2019a,b) also reported greater energy values for HPDG and WFS when included in finishing diets at 20 to 40% of DM compared with dent corn. When WFS was included at 15 and 45% in the finishing diet for Zebu cattle in Brazil, its feeding values were 13.46 and 13.3% greater, respectively, relative to the replaced blends of ground flint corn, citrus pulp, soybean meal, and urea (Lima Jr. et al., 2019).

In the current study, as hypothesized, when HPDG was included in the diet as a protein supplement, at 10.3% of diet DM, it was as effective as the blend of whole cottonseed and soybean meal (CON), with no differences in cattle DMI, growth performance, and carcass traits. When fed at low levels of inclusion, HPDG presented similar energy value as the blend of whole cottonseed plus soybean meal. These performance results were corroborated by the metabolism experiment, where no effects in DM and nutrient digestibility were observed between the CON and HPDG. On the other hand, when HPDG was included at 20 and 40% of diet DM in Garland et al. (2019b,c) trials, DM digestibility was decreased, but gross energy intake, diet energy content, and cattle growth performance were greater than for the control diet. In the current study, compared with the CON, feeding HPDG improved rumen fermentation, decreasing the molar proportion of acetate and increasing the molar proportion of propionate; thus, reducing acetate:propionate ratio, and decreasing ruminal NH_3_-N due to the lower RDP (40.0%, % of CP) in this byproduct compared to whole cottonseed and soybean meal. However, these improvements in rumen fermentation and protein metabolism apparently were not enough to improve cattle growth performance.

In contrast to what is consistently reported in the literature when WDGS replaced dent corn in US feedlot diets (Klopfenstein et al., 2008; Bremer et al., 2011; DiConstanzo et al., 2015; NASEM, 2016), in our study, WFS did not improve cattle ADG, final BW, or HCW when it replaced the blend of ground flint corn, whole cottonseed, and soybean meal in the control diet. The metabolism study corroborates that there was no difference in cattle performance because DM intake and nutrient digestibility and rumen VFA profiles were not different between the CON and the WFS diets. It is important to note that the WFS used in this study contained only 18.2% CP versus contents greater than 30% reported for the traditional WDGS (Klopfenstein et al., 2008; Bremer et al., 2011; DiConstanzo et al., 2015; NASEM, 2016). It has been hypothesized that the greater energy content of WDGS, MDGS, and DDGS relative to dent corn may be partially related to the high contents of fat and rumen undegradable protein (RUP) of these byproducts (Carlson et al., 2016). However, in the present study, feeding WFS did not affect DMI in the growth performance study, and a significant decrease in DMI was observed in the metabolism study. Despite being a numerical difference, feeding 30% WFS with 18.2% CP in the diet resulted in 7.19% greater G:F and 6.0 and 6.9% greater observed NE_m_ and NE_g_, respectively, compared with CON. The feeding value of the WFS was 24%, and the NE_g_ was 23% greater than the replaced blend of ground flint corn, whole cottonseed, and soybean meal.

Lima Jr. et al. (2019) fed increasing levels of WFS containing 33.85% CP in a typical southeast Brazilian finishing diet containing 0 or 3% corn oil, ground flint corn, citrus pulp, soybean meal, and urea, and also found no differences in ADG and HCW, but a linear decrease in DMI when corn oil was not fed versus a quadratic decrease when corn oil was fed. The authors also discussed that feeding 15 and 45% of WFS in the finishing diet increased G:F by 5.2 and 10.4%, NE_m_ by 3.6 to 9.84%, and NE_g_ by 6.29% to 14.17%. The feeding values reported for WFS were 34.6 and 33.0% greater, respectively, compared with the replaced blends of ground flint corn, citrus pulp, soybean meal, and urea. On the other hand, Ferreira et al. (2020) fed increasing levels of WFS (0, 15, 30, and 45%) for finishing cattle fed a control diet (0% WFS) based on flint corn and soybean meal. Feeding WFS tended to increase cattle DMI and ADG quadratically with no effects on cattle feed efficiency and observed NE content of the diets. Garland et al. (2019a,c) reported similar DMI, greater ADG, G:F, and HCW for cattle that received diets containing 40% (% DM) WFS compared with a high dent corn (50% dry rolled:50% high moisture) control diet and 26% greater feeding value for WFS compared with dent corn plus urea. However, DM and organic matter (OM) digestibilities were less for diets containing WFS than dent corn. On the other hand, GE content (Mcal/kg DM) was greater for the WFS, and digestible energy (DE) intake tended to be greater for cattle that received WFS diets compared with the high corn control diet. In our study, GE content of the DFS and WFS were respectively 5.34 and 5.40 Mcal/kg DM compared with values of 5.45, 4.51, and 4.34 Mcal/kg DM for whole cottonseed, ground flint corn, and soybean meal, respectively.

The results from our study may differ from previous literature in part due to differences in corn kernel makeup. For instance, the CON diet was not a typical high-dent corn diet, as reported in the studies reviewed by Klopfenstein et al. (2008) and by Bremer et al. (2011). Instead, the CON diet contained ground flint corn, whole cottonseed, soybean meal, and soybean hulls, a typical diet of feedlots located in the western areas of Brazil (Pinto and Millen, 2018). Considering the lower net energy content of flint corn compared with dent corn (Correa et al., 2002; Gouvêa et al., 2016; Marques et al., 2016), one could expect a greater response to WFS than reported in the US literature with dent corn. This expectation is based on the fact that flint corn has a harder, more vitreous endosperm, which reduces starch availability in the rumen. As a result, the additional fermentable fiber and residual energy provided by WFS could have a relatively larger impact on flint corn-based diets than in dent corn-based diets, where energy from starch is more readily available. On the other hand, the control diet contained whole cottonseed, which increases the net energy density of the diets based on flint corn (NASEM, 2016). All diets contained 12.0% soybean hulls, which increased the energy density of diets with 40% WDGS and dent corn (Bittner et al., 2016) when included at 12.5% DM.

The 10 to 12 kg greater HCW for bulls fed DFS compared with the other treatments is a result of greater ADG and final BW. Bulls fed DFS tended to have greater DMI than those fed CON. Furthermore, steers fed DFS had a more efficient rumen fermentation than those fed CON, with a lower molar proportion of acetate and greater propionate. When rumen fermentation is changed towards more propionate, less methane is produced, and more dietary energy is retained by the animal (NASEM, 2016). On the other hand, steers fed DFS had less DM digestibility than CON. When the coefficients of DM digestibility from the metabolism study are applied to the DMI observed in the performance experiment, cattle fed DFS consumed less digestible DM than CON (data not shown). It appears that DM digestibility and intake of digestible DM are not good predictors of cattle performance when corn ethanol byproducts are fed, as highlighted by Wilson et al. (2021). This negative effect of corn ethanol byproducts on diet digestibility was also reported by Garland et al. (2019c) despite their improvement in cattle performance (Garland et al., 2019a, b).

When corn ethanol byproducts replace corn in the diet, displacing starch, the risk of rumen acidosis may decrease in feedlot cattle (DiConstanzo et al., 2015; NASEM, 2016). However, Garland et al. (2019c) reported that minimum, average, and maximum rumen pH were not affected when blends of dry rolled and high moisture dent corn were replaced by HPDG (20% diet DM), WFS, DDGS, and WDGS (40% of diet DM). The minimum, average, and maximum rumen pH for the high corn control diet was 4.89, 5.40, and 6.08, respectively. In our study, the average rumen pH varied from 6.59 to 6.65 and was not affected by treatments. The values for rumen pH in the current study were considerably greater than the ones reported by Garland et al. (2019c) probably due to the low starch degradation in the rumen of ground flint corn compared to dent corn (Correa et al. 2002), the starch dilution effect with the inclusion of whole cottonseed and soybean hulls in the control diet, and the high content of NDF of whole cottonseed (NASEM, 2016). The low DMI of cannulated steers in the metabolism experiment (average 1.75% of BW) also contributed to the greater rumen pH observed in the current study.

In summary, incorporating high protein distillers grain (HPDG) at a modest level (10.3% of dry matter) in finishing diets that include flint corn offers a viable protein supplement alternative to traditional sources like whole cottonseed and soybean meal. Furthermore, feeding corn ethanol byproducts, wet fiber plus solubles (WFS) and dry fiber plus solubles (DFS), at 30% of dry matter in finishing diets, leads to improved cattle performance likely due to both the partial displacement of flint corn and the complete replacement of whole cottonseed and soybean meal.

Despite these positive outcomes, further investigations are essential to ascertain the energy content of DFS and WFS in comparison to blends containing flint corn and conventional protein sources. Additional studies to evaluate the energy value of DFS and WFS will contribute valuable insights into optimizing the formulation of finishing diets for enhanced cattle productivity and overall nutritional efficiency, particularly if processes change or are adjusted during fractionation of distillers grains from ethanol production.
